# Docetaxel for Nonmetastatic Prostate Cancer: Long-Term Survival Outcomes in the STAMPEDE Randomized Controlled Trial

**DOI:** 10.1093/jncics/pkac043

**Published:** 2022-07-25

**Authors:** Nicholas D James, Fiona C Ingleby, Noel W Clarke, Claire L Amos, Gerhardt Attard, Christopher D Brawley, Simon Chowdhury, William Cross, David P Dearnaley, Duncan C Gilbert, Silke Gillessen, Robert J Jones, Ruth E Langley, Archie Macnair, Zafar I Malik, Malcolm D Mason, David J Matheson, Robin Millman, Chris C Parker, Hannah L Rush, J Martin Russell, Carly Au, Alastair W S Ritchie, Ricardo Pereira Mestre, Imtiaz Ahmed, Alison J Birtle, Susannah J Brock, Prantik Das, Victoria A Ford, Emma K Gray, Robert J Hughes, Caroline B Manetta, Duncan B McLaren, Ashok D Nikapota, Joe M O’Sullivan, Carla Perna, Clive Peedell, Andrew S Protheroe, Santhanam Sundar, Jacob S Tanguay, Shaun P Tolan, John Wagstaff, Jan B Wallace, James P Wylie, Anjali Zarkar, Mahesh K B Parmar, Matthew R Sydes

**Affiliations:** Division of Radiotherapy and Imaging, The Institute of Cancer Research and Royal Marsden NHS Foundation Trust, London, UK; MRC Clinical Trials Unit at University College London (UCL), Institute of Clinical Trials and Methodology, UCL, London, UK; The Christie and Salford Royal Hospitals, Manchester, UK; MRC Clinical Trials Unit at University College London (UCL), Institute of Clinical Trials and Methodology, UCL, London, UK; UCL Cancer Institute, London, UK; MRC Clinical Trials Unit at University College London (UCL), Institute of Clinical Trials and Methodology, UCL, London, UK; Guy’s and St. Thomas’ NHS Foundation Trust, London, UK; Sarah Cannon Research Institute, London, UK; St James’s University Hospital, Leeds, UK; Division of Radiotherapy and Imaging, The Institute of Cancer Research and Royal Marsden NHS Foundation Trust, London, UK; MRC Clinical Trials Unit at University College London (UCL), Institute of Clinical Trials and Methodology, UCL, London, UK; Istituto Oncologico della Svizzera Italiana, Bellinzona, Switzerland; Institute of Cancer Sciences, University of Glasgow, Beatson West of Scotland Cancer Centre, Glasgow, UK; MRC Clinical Trials Unit at University College London (UCL), Institute of Clinical Trials and Methodology, UCL, London, UK; MRC Clinical Trials Unit at University College London (UCL), Institute of Clinical Trials and Methodology, UCL, London, UK; Guy’s and St. Thomas’ NHS Foundation Trust, London, UK; The Clatterbridge Cancer Centre NHS Foundation Trust, Bebington, UK; School of Medicine, Cardiff University, Cardiff, UK; Faculty of Education, Health and Wellbeing, University of Wolverhampton, Wolverhampton, UK; MRC Clinical Trials Unit at University College London (UCL), Institute of Clinical Trials and Methodology, UCL, London, UK; Division of Radiotherapy and Imaging, The Institute of Cancer Research and Royal Marsden NHS Foundation Trust, London, UK; MRC Clinical Trials Unit at University College London (UCL), Institute of Clinical Trials and Methodology, UCL, London, UK; Guy’s and St. Thomas’ NHS Foundation Trust, London, UK; Institute of Cancer Sciences, University of Glasgow, Beatson West of Scotland Cancer Centre, Glasgow, UK; MRC Clinical Trials Unit at University College London (UCL), Institute of Clinical Trials and Methodology, UCL, London, UK; Urology Department, Gloucestershire Royal NHS Foundation Trust, Gloucester, UK (retired); Oncology Institute of Southern Switzerland, Bellinzona, Switzerland; Institute of Oncology Research (IOR), Bellinzona, Switzerland; Southend University NHS Trust, Southend, UK; Rosemere Cancer Centre Lancs Teaching Hospitals, Preston, UK; University of Manchester, Manchester, UK; University of Central Lancashire (UCLan), Lancaster, UK; University Hospital Dorset, Cardiff, UK; University Hospitals of Derby NHS Foundation Trust, Derby, UK; Royal Devon and Exeter NHS Foundation Trust, Exeter, UK; Musgrove Park Hospital, Taunton, UK; Mount Vernon Cancer Centre, Cardiff, UK; Sussex Cancer Centre, University Hospitals Sussex, Brighton, UK; Edinburgh Cancer Centre, Western General Hospital, Edinburgh, UK; Sussex Cancer Centre, University Hospitals Sussex, Brighton, UK; Worthing and Southlands Hospital, Worthing, UK; Patrick G. Johnston Centre for Cancer Research, Queen’s University Belfast, Belfast, UK; Royal Surrey NHS Foundation Trust, Guildford, UK; James Cook University Hospital, Middlesbrough, UK; Oxford University Hospitals NHS Foundation Trust, Oxford, UK; Nottingham University Hospitals NHS Trust, Nottingham, UK; Velindre Cancer Centre, Cardiff, UK; The Clatterbridge Cancer Centre NHS Foundation Trust, Bebington, UK; Swansea University College of Medicine & The South West Wales Cancer Centre, Swansea, UK; Beatson West of Scotland Cancer Centre, Glasgow, UK; The Christie NHS Foundation Trust, Manchester, UK; University Hospitals Birmingham, UK; MRC Clinical Trials Unit at University College London (UCL), Institute of Clinical Trials and Methodology, UCL, London, UK; MRC Clinical Trials Unit at University College London (UCL), Institute of Clinical Trials and Methodology, UCL, London, UK

## Abstract

**Background:**

STAMPEDE previously reported adding upfront docetaxel improved overall survival for prostate cancer patients starting long-term androgen deprivation therapy. We report long-term results for non-metastatic patients using, as primary outcome, metastatic progression-free survival (mPFS), an externally demonstrated surrogate for overall survival.

**Methods:**

Standard of care (SOC) was androgen deprivation therapy with or without radical prostate radiotherapy. A total of 460 SOC and 230 SOC plus docetaxel were randomly assigned 2:1. Standard survival methods and intention to treat were used. Treatment effect estimates were summarized from adjusted Cox regression models, switching to restricted mean survival time if non-proportional hazards. mPFS (new metastases, skeletal-related events, or prostate cancer death) had 70% power (α = 0.05) for a hazard ratio (HR) of 0.70. Secondary outcome measures included overall survival, failure-free survival (FFS), and progression-free survival (PFS: mPFS, locoregional progression).

**Results:**

Median follow-up was 6.5 years with 142 mPFS events on SOC (3 year and 54% increases over previous report). There was no good evidence of an advantage to SOC plus docetaxel on mPFS (HR = 0.89, 95% confidence interval [CI] = 0.66 to 1.19; *P* = .43); with 5-year mPFS 82% (95% CI = 78% to 87%) SOC plus docetaxel vs 77% (95% CI = 73% to 81%) SOC. Secondary outcomes showed evidence SOC plus docetaxel improved FFS (HR = 0.70, 95% CI = 0.55 to 0.88; *P* = .002) and PFS (nonproportional *P* = .03, restricted mean survival time difference = 5.8 months, 95% CI = 0.5 to 11.2; *P* = .03) but no good evidence of overall survival benefit (125 SOC deaths; HR = 0.88, 95% CI = 0.64 to 1.21; *P* = .44). There was no evidence SOC plus docetaxel increased late toxicity: post 1 year, 29% SOC and 30% SOC plus docetaxel grade 3-5 toxicity.

**Conclusions:**

There is robust evidence that SOC plus docetaxel improved FFS and PFS (previously shown to increase quality-adjusted life-years), without excess late toxicity, which did not translate into benefit for longer-term outcomes. This may influence patient management in individual cases.

STAMPEDE’s “docetaxel comparison” previously showed a clear, clinically important overall survival advantage for adding upfront docetaxel across men with locally advanced or metastatic prostate cancer initiating long-term androgen deprivation therapy (ADT) (1). Trials of docetaxel in this setting reported before STAMPEDE showed inconsistent results in locally advanced disease: Upfront docetaxel improved relapse-free survival (survival not reported) in nonmetastatic disease in GETUG-12 (2); improved failure-free survival (FFS) and progression-free survival (PFS) in metastatic disease without evidence of improvement in overall survival in GETUG-15’s primary and long-term analyses (3,4); and a survival benefit in metastatic disease in CHAARTED’s early released and long-term analyses (5,6). The prospectively planned STOPCAP meta-analysis, published alongside STAMPEDE, showed substantial, reliable evidence that upfront docetaxel improved survival for men with metastatic disease (7). Nonmetastatic patients have considerably better prognosis than metastatic patients, and despite clear evidence of improved FFS from upfront docetaxel, there was insufficient evidence on overall survival because of the low number of events (7).

International guidelines incorporate upfront docetaxel into recommendations for suitable patients with metastatic prostate cancer, particularly high-volume disease (8,9). Metastatic-dependent guidelines reflect separate clinical considerations because stratification of patients increasingly drives treatment decisions. Therefore, the STAMPEDE Trial Management Group felt it appropriate to report separately long-term results from the metastatic and nonmetastatic patients. Long-term metastatic group results confirmed a survival advantage with upfront docetaxel with a hazard ratio (HR) of 0.81 (95% confidence interval [CI] = 0.69 to 0.95) (10).

We report long-term analysis of patients with nonmetastatic disease, allowing in-depth assessment of outcomes of docetaxel in node-positive (N+) and node-negative (N0) populations and by use of standard-of-care (SOC) prostate radiotherapy. The nonmetastatic patients STAMPEDE’s docetaxel comparison control arm previously demonstrated 96% 2-year survival (11). With such a low event rate, powering comparisons based on survival with traditional relative treatment effects is not feasible. The ICECaP consortium showed metastasis-based outcome measures as an acceptable surrogate for survival-based outcome measures (12), so these analyses focus on metastatic progression-free survival (mPFS) with good power and long-term follow-up.

## Methods

### Design

STAMPEDE uses a multi-arm multistage platform design to compare treatments against SOC (13). Patients with prostate cancer were recruited to the docetaxel comparison from 119 sites in the United Kingdom and Switzerland between Octocter 5, 2005, and March 31, 2013. Eligibility was newly diagnosed prostate cancer or high-risk relapse after previous radical treatment without previous long-term ADT. Good clinical practice guidelines were followed, with the necessary regulatory and ethical approvals in place.

All patients were planned for long-term ADT as the basis for SOC. Here, the relevant patients were randomly assigned 2:1 to control (SOC) or research: SOC plus upfront docetaxel. Random assignment used minimization with a 20% random element, stratified by age at randomization (younger than 70 years vs 70 years and older), World Health Organization (WHO) performance score (0 vs 1 or 2), baseline metastases (yes or no) and nodal status (negative, positive, or unspecified), planned ADT type, use of aspirin or other nonsteroidal anti-inflammatory drugs, participating hospital/site, and from 2011, planned radiotherapy. This algorithm was developed and used centrally at MRC Clinical Trials Unit at University College London.

### Procedures

Full details for administering docetaxel were reported previously (1). In summary, following written informed consent, patients randomly assigned to research arm had 6 docetaxel cycles (75 mg/cm^2^) 3 weekly plus 10 mg prednisolone daily in addition to SOC ADT. For patients without a contraindication, SOC could include prostate radiotherapy; before November 14, 2011, irrespective of nodal status, such SOC radiotherapy was optional but encouraged; from November 14, 2014, such SOC radiotherapy was mandated for patients with N0 disease and encouraged for N+ disease; this change was to implement the findings of the MRC PR07/NCIC PR.3 and SPCG-7 trials (14,15). Planned use of radiotherapy was reported prior to random assignment.

Information on any adverse events (AEs) or disease progression was reported at routine follow-up visits scheduled in the protocol to be 6 weekly in the first 6 months postrandomization, then 12 weekly until 2 years, 6 monthly until 5 years, and annually thereafter. AEs were classified and graded following the National Cancer Institute Common Terminology Criteria for Adverse Events v4.0.

### Outcomes

The first findings of the docetaxel comparison were reported previously based on a May 13, 2015, data freeze (1). Data here were frozen on July 13, 2018. The statistical analysis plan specified that long-term efficacy analyses of the nonmetastatic (M0) cohort would be reported separately from the metastatic (M1) patient cohort (10). The main focus for this analysis was mPFS, shown to be a surrogate measure for overall survival in M0 patient cohorts (12). mPFS was defined as time from randomization to new metastatic disease or death from prostate cancer. Secondary outcomes included FFS (time to biochemical progression, lymph node progression, distant metastatic progression, or prostate cancer death), PFS (time to the first FFS event, excluding biochemical progression), overall survival (time to death from any cause), and prostate cancer–specific survival (PCSS; time to prostate cancer death). Prostate-specific antigen (PSA) was measured at each follow-up assessment until disease progression. Biochemical progression was defined as a rise to at least 4 ng/mL or 50% above the nadir attained within 24 weeks after randomization. The PSA nadir could not be calculated if PSA did not decrease after randomization, so biochemical progression, in those instances, was taken as the date of randomization; this applied to few patients (<1%).

Cause of death was categorized algorithmically to differentiate prostate cancer and non-prostate cancer causes, with rules agreed to in 2018 by the Trial Management Group (Supplementary Box 1, available online). Each death not algorithmically assignable was clinically reviewed.

Patients without the event of interest reported were censored at their latest time event free.

### Statistical Analysis

In brief, the comparison’s sample size targeted a hazard ratio of 0.75 for overall survival, requiring approximately 400 control arm deaths across M0 and M1 patients (1). This long-term efficacy analysis in M0 disease was scheduled for approximately 3 years after the initial analysis, by when 50% more mPFS events were projected, allowing for approximately 55% power to detect a hazard ratio of 0.75 or 70% power for a hazard ratio of 0.70 for mPFS.

For efficacy analyses, patients were included under their allocated treatment group, as per intention-to-treat principles. For safety analyses, patients were analyzed in groups according to treatment received: the control-safety group included patients allocated to SOC and 18 research patients not reported as starting docetaxel (n = 478); the docetaxel-safety group consisted of 212 research arm patients who reported starting trial treatment.

Standard survival analysis methods in Stata v15.1 were used for time-to-event analyses. Follow-up duration was estimated using reverse-censored Kaplan-Meier on death. Treatment efficacy was interpreted from a hazard ratio and median time-to-event estimated from Cox proportional hazards regression models, stratified for minimization factors as used at randomization (nodal stage, age at randomization, WHO performance score, use of aspirin or nonsteroidal anti-inflammatory drugs, planned use of SOC radiotherapy), except for participating hospital. Time period was included as stratification in the models to delineate periods of STAMPEDE, where other trial arms were opened or closed to recruitment or with SOC change of practice. Nonparametric stratified log-rank tests were used to test differences between trial arms. Flexible parametric models (16,17) were fitted to estimate 5-year survival using (5,5) degrees of freedom and stratified as per the Cox models. Cox models were tested for evidence of nonproportional hazards and, if required, treatment efficacy then emphasized restricted mean survival time (RMST) with t* = 108 months (18). Competing risks regression analysis techniques were used to analyze PCSS (nonprostate cancer death as a competing event) (19). Statistical tests were 2-sided; 95% confidence intervals and *P* values are reported. Kaplan-Meier graphs have been presented using the KMunicate format (20).

Exploratory subgroup analyses are presented for the primary outcome to assess consistency of docetaxel treatment effect across baseline factors (nodal status, Gleason score, age at randomization, WHO performance score, and recurrent disease status).

**Figure 1.**
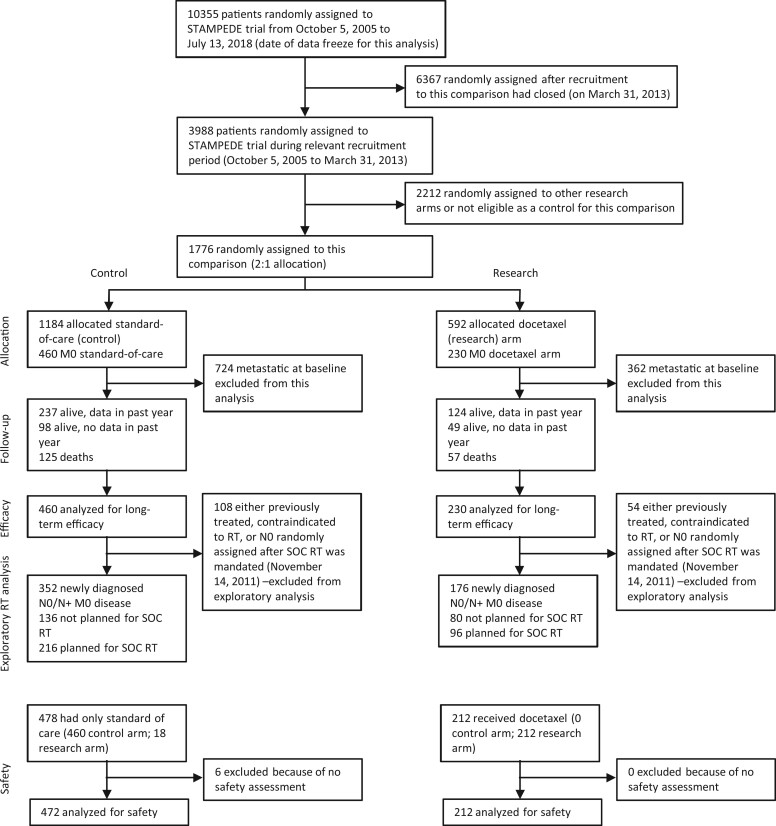
CONSORT diagram. M0 = nonmetastatic; N+ = node positive; N0 = node negative; SOC = standard of care; RT = radiotherapy;

Further exploratory analyses assessed the efficacy of SOC radiotherapy for each outcome measure. These analyses focused on patients with no contraindication to radiotherapy and with either N0 disease recruited before SOC November 14, 2011 (before SOC radiotherapy was mandated) or N+ disease recruited any time (see Figure 1). These analyses, regardless of treatment allocated in the docetaxel comparison, build on previous analyses that had included only control group patients (21). The analysis principles followed those specified above for time-to-event analyses but focused on comparing patients who did not report preplanned SOC radiotherapy to those who did. Models were stratified by treatment allocated in the randomized docetaxel comparison in addition to the stratification factors specified above. Subgroup analyses explored the consistency of the effect of SOC radiotherapy across nodal status (N0 vs N+) as well as across trial arm (control vs docetaxel).

### Context

To contextualize the findings, estimates of treatment effects on overall survival from relevant trials were combined using standard meta-analysis methods. The inclusion criteria were randomized trial; nonmetastatic prostate cancer; control treatment of long-term hormone therapy with or without prostate radiotherapy; and with survival data published.

## Results

There were 690 nonmetastatic patients recruited to STAMPEDE’s docetaxel comparison between October 5, 2005, and March 31, 2013: 460 patients to the control group and 230 patients to the docetaxel group. Figure 1 details patient numbers and inclusion in each analysis. The data for this updated efficacy analysis were frozen on July 13, 2018, and as previously reported, baseline patient characteristics were well balanced across control and treatment groups (Table 1). Of the 230 patients, 18 (8%) allocated to docetaxel group did not report starting docetaxel. The median duration of follow-up was 81.2 months (quartiles 63.2 and 99.7), which was consistent across both the control (81.6 months, quartiles 62.2 and 100.8) and docetaxel groups (78.3 months, quartiles 63.8-97.9).

**Table 1.** Baseline characteristics by trial arm^a^

Patient characteristic	Control	Docetaxel
	No. (%)	No. (%)
Randomization, 2:1 allocation	460 (100)	230 (100)
Median age at randomization (IQR), y	65 (61-70)	66 (61-71)
WHO performance status		
0	401 (87)	191 (83)
1-2	59 (13)	39 (17)
T stage		
0	4 (1)	1 (<1)
1	9 (2)	0 (0)
2	38 (8)	9 (4)
3	352 (77)	193 (84)
4	48 (10)	23 (10)
Unreported	9 (2)	4 (2)
Nodal status		
0	280 (61)	141 (61)
Positive	178 (39)	88 (38)
Unreported	2 (<1)	1 (<1)
Gleason score		
≤7	124 (27)	45 (20)
8-10	331 (72)	184 (80)
Unreported	5 (1)	1 (<1)
Median PSA (IQR), ng/mL	42 (17-87)	44 (19-93)
Median time from diagnosis to randomization (IQR), d	81 (61-110)	79 (60-104)
Planned SOC radiotherapy		
Not planned	170 (37)	92 (40)
Planned	290 (63)	138 (60)
Previously treated		
No	427 (93)	217 (94)
Yes	33 (7)	13 (6)
Pain from prostate cancer		
Absent	432 (94)	221 (96)
Present	26 (6)	8 (4)
Unknown	2 (<1)	1 (<1)
Year of randomization		
2005	2 (<1)	1 (<1)
2006	15 (3)	7 (3)
2007	33 (7)	17 (7)
2008	49 (11)	25 (11)
2009	56 (12)	29 (13)
2010	70 (15)	32 (14)
2011	99 (22)	49 (21)
2012	105 (23)	53 (23)
2013	31 (7)	17 (7)
Total	460 (100)	230 (100)

a IQR = interquartile range; SOC = standard of care; WHO = World Health Organization.

**Figure 2.**
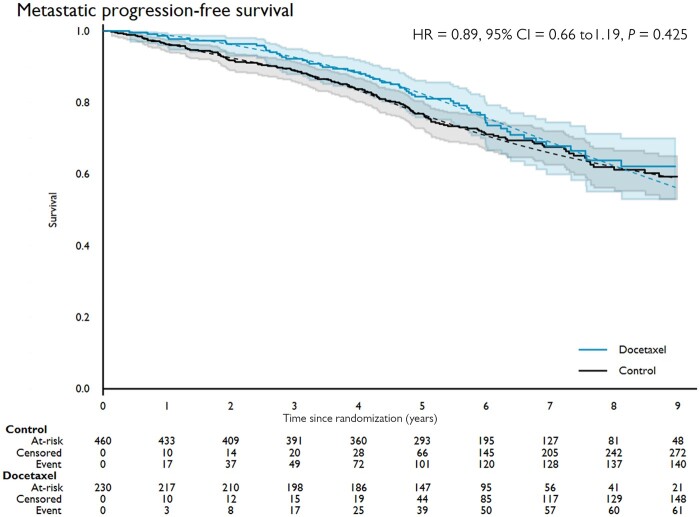
Metastatic progression-free survival by allocated treatment. Kaplan-Meier curves (**solid line**) and fitted flexible parametric model estimates (**dashed line**) for metastatic progression-free survival, by trial arm (hazard ratio [HR] = 0.89, 95% confidence interval [CI] = 0.66 to 1.19; *P* = .425).

For the primary outcome measure for this long-term analysis, metastatic mPFS, there were 207 mPFS events reported: 142 of 460 (31%) control group and 65 of 230 (28%) docetaxel group. There was no good evidence that docetaxel improved survival (HR = 0.89, 95% CI = 0.66 to 1.19; stratified log-rank test *P* = .43; Figure 2). There was no evidence (*P* = .23) of nonproportional hazards in the treatment effect on mPFS. The proportion event free at 5 years was 77% (95% CI = 73% to 81%) in the control and 82% (95% CI = 78% to 87%) in the docetaxel group.

Exploratory subgroup analyses looked at consistency of docetaxel’s effect on mPFS across baseline characteristics of interest (including nodal status, Gleason score, age at randomization, WHO performance score, and recurrent disease) and found no evidence of inconsistency in the effect in the groups examined (Supplementary Figure 1, available online).

**Figure 3.**
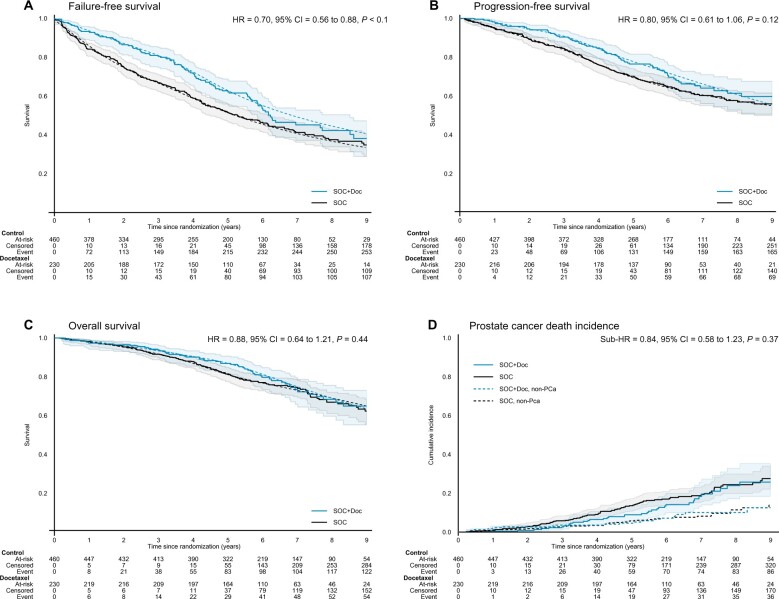
Other efficacy outcome measures by allocated treatment. Kaplan-Meier curves (**solid line**) and fitted flexible parametric model estimates (**dashed line**), by trial arm, for **(A)** failure-free survival; **(B)** progression-free survival; and **(C)** overall survival. **D)** shows the cumulative incidence function, by trial arm, for prostate cancer death (**solid line**) and nonprostate cancer death (**dashed line**). CI = confidence interval; HR = hazard ratio.

There was clear evidence of benefit with docetaxel on FFS (HR = 0.70, 95% CI = 0.56 to 0.88; *P* = .002; Figure 3, A) and improved PFS with an increase in RMST over 108 months of 5.8 months (95% CI = 1.2 to 10.5; *P* = .015; Figure 3, B); a hazard ratio of 0.80 (95% CI = 0.61 to 1.06; *P* = .12) was impacted by evidence of nonproportional hazards of treatment effect (*P* = .03). However, these earlier improvements did not translate into improvements in overall survival or PCSS. There were 182 deaths with 88 of 125 (70%) in the control group and 39 of 57 (68%) in the docetaxel group attributed to prostate cancer on review. The treatment effect for overall survival was estimated as a hazard ratio of 0.88 (95% CI = 0.64 to 1.21; *P* = .44; Figure 3, C) with 5-year survival of 81% (95% CI = 77% to 85%) and 87% (95% CI = 82% to 91%) for the control and docetaxel groups, respectively, and for PCSS (sub-HR = 0.84, 95% CI = 0.58 to 1.23; *P* = .34; Figure 3, D). The hazard ratio, 5-year survival, and RMST for each outcome measure is summarized in Supplementary Table 2 and Supplementary Figure 2 (available online).

The worst grade AE in the first year of follow-up postrandomization was higher with docetaxel: 15% control-safety group reported grade 3-5 AEs compared with 36% docetaxel-safety group (Table 2). There was no good evidence of differences in the worst grade of AEs subsequent to the initial year after randomization: 28% control-safety group reported grade 3-5 AE vs 30% docetaxel-safety group.

**Table 2.** Worst toxicity grade reported per patient (across all CTCAE categories) for 1) up to 1 year on the trial and 2) after 1 year on the trial^a^

Worst toxicity grade	Up to 1 year^b^		After 1 year^b^	
	Control	Docetaxel	Control	Docetaxel
	No. (%)	No. (%)	No. (%)	No. (%)
0	11 (2)	2 (1)	7 (2)	6 (3)
1	170 (36)	54 (25)	130 (30)	48 (24)
2	218 (46)	80 (38)	171 (40)	87 (43)
3	67 (14)	44 (21)	104 (24)	50 (25)
4	5 (1)	29 (14)	17 (4)	11 (5)
5	1 (<1)	3 (1)	1 (<1)	0 (0)
No FU/SAE reported	6 (N/A)	0 (N/A)	6 (N/A)	0 (N/A)
Not on FU after 1 year	N/A	N/A	42 (N/A)	10 (N/A)
Total^c^	478 (100)	212 (100)	478 (100)	212 (100)

a Further details are shown in Supplementary Table 6 (available online). CTCAE = Common Terminology Criteria for Adverse Events; FU = Follow-up; N/A = not applicable; SAE = Serious Adverse Event.

b Timed from randomization.

c Total numbers shown for safety population, where 18 patients allocated to the docetaxel group never started docetaxel treatment and are therefore included in the standard-of-care group for safety reporting. Note that “N/A” data refers to patients who did not report toxicity data after this point (either died or withdrawn from the trial or not reporting toxicity after disease progression as specified in the trial protocol).

Supplementary Table 1 (available online) shows evidence of at least 1 subsequent therapy following progression for control group patients (41%) compared with docetaxel group patients (34%) and of different patterns of reported subsequent therapy by group.

**Figure 4.**
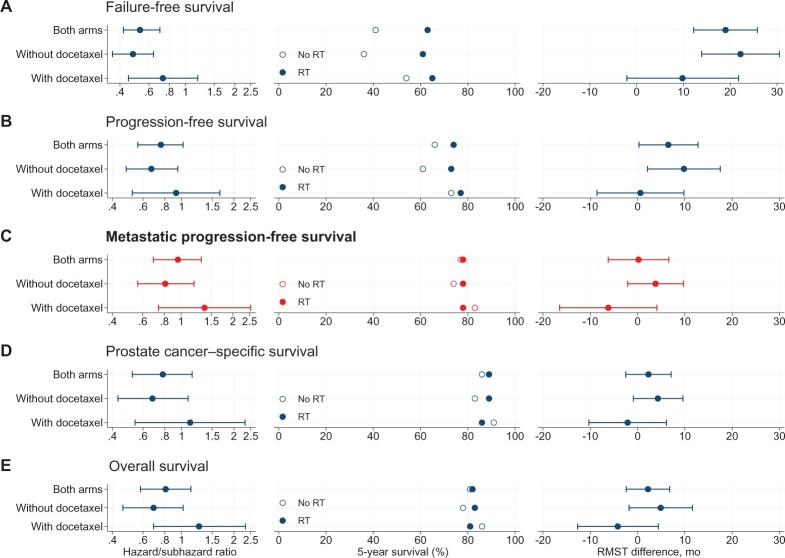
Effect of standard-of-care (SOC) radiotherapy (RT), with or without docetaxel treatment. Results are shown for **(A)** failure-free survival, **(B)** progression-free survival, **(C)** metastatic progression-free survival (primary outcome), **(D)** overall survival, and **(E)** prostate cancer–specific survival. **Left:** Hazard/subhazard ratio with 95% confidence interval. **Center:** 5-year survival estimates, by arm. **Right: **Difference (RMST estimate) between SOC RT groups in survival time, where a positive difference indicates longer survival time for the subgroup planned for RT. RMST = restricted mean survival time.

Further exploratory analyses examined the impact of SOC radiotherapy in a subset of 528 of 690 (77%) patients, regardless of treatment allocation, for whom the use of SOC radiotherapy was optional (Figure 1), and the number of patients in each subgroup (by nodal status and trial group) is shown in Supplementary Table 3 (available online). There was clear evidence of a benefit of SOC radiotherapy on FFS overall (HR = 0.53, 95% CI = 0.42 to 0.68; *P* < .001) and in the N0 and N+ subgroups (Supplementary Table 4 and Supplementary Figure 3, B, available online). There was some evidence that SOC radiotherapy also improved PFS overall (HR = 0.76, 95% CI = 0.56 to 1.02; *P* = .065; Supplementary Figure 3, C, available online). However, there was no good evidence of a benefit with SOC radiotherapy in terms of mPFS (HR = 0.96, 95% CI = 0.69 to 1.31; *P* = .78; Supplementary Figure 3, A and C, available online), overall survival (HR = 0.81, 95% CI = 0.58 to 1.13; *P* = .21; Supplementary Figure 3, C and D, available online), or PCSS (sub-HR = 0.78, 95% CI = 0.52 to 1.15; *P* = .21; Supplementary Figure 3, C and E, available online). Figure 4 and Supplementary Table 5 (available online) show evidence of a benefit of SOC radiotherapy in the control group on all outcome measures but, with the exception of FFS, no good evidence of any benefit to SOC radiotherapy in the docetaxel group.

Three trials met the criteria for a combined analysis with these STAMPEDE data: GETUG-12, RTOG-0521, and ARTIC AOM 03108 (Table 3; Supplementary Figure 4, available online). Together, these 4 trials have reported 461 deaths in 1978 randomly assigned, evaluable patients. The combined hazard ratio was 0.84 (95% CI = 0.69 to 1.02; *P* = .08) with no evidence of heterogeneity of effect across the trials (*I*^2^ = 0.0).

**Table 3.** Combined analysis of eligible trials

Trial	Published	Evaluable	Control	Research	HR (95% CI)
GETUG-12	2015	413/413	49/206	42/207	0.94 (0.60 to 1.49)
RTOG 0521	2019	563/621	59/281	43/282	0.69 (0.46 to 1.04)^a^
ARTIC AOM 03108	2019	250/254	46/125	40/125	0.86 (0.56 to 1.31)
STAMPEDE M0 (SOC +/- Doc)	2016	690/690	65/460	31/230	0.95 (0.62 to 1.46)
STAMPEDE M0 (SOC +/- Doc)	2021	690/690	125/460	57/230	0.88 (0.64 to 1.21)
Combined		1916/1978	279/1072	182/844	0.84 (0.69 to 1.02)

a Presented as 90% confidence interval (CI) around 0.69 (0.45 to 0.97). DOC = docetaxel; HR = hazard ratio; SOC = standard of care.

## Discussion

Our updated results from STAMPEDE’s “docetaxel comparison” for patients with nonmetastatic prostate cancer starting long-term hormone therapy, with 3 additional years of follow-up, extending median follow-up to approximately 6.5 years, show good evidence that docetaxel improved FFS and PFS, but this did not translate into meaningful improvements in key long-term efficacy outcome measures, mPFS, overall, or PCSS.

Proponents of early chemotherapy may argue this comparison, which was not explicitly designed with traditional levels of power to detect a difference in nonmetastatic patients, show longer median mPFS (87.1 months vs 90.4 months) and higher 5-year survival (81% vs 87%) with docetaxel. They may also argue there was insufficient patient numbers or that our dataset is still too immature to detect any potential benefit of docetaxel in a population with good outcomes. The trial team has closed out site follow-up for these patients, but most patients consented to access to data through national registries, which could allow for longer-term assessment.

Sceptics may argue there was insufficient power on mPFS to detect a clinically meaningful benefit had there been one. They may also argue, from ICECaP’s surrogacy work (12), it is unlikely a meaningful improvement in long-term survival would emerge with continued follow-up given the observed modest impact on mPFS. Nearly three-quarters of patients were still alive when this dataset was frozen, with median age of survivors approaching 75 years. Only three-tenths of reported deaths had been attributed to causes other than prostate cancer, but deaths from competing causes are likely to become more common in subsequent years, which would impact the ability to detect any PCSS effect.

We previously reported higher rates of AEs in the docetaxel group. Here, we show the AE rate after the first year of follow-up, starting about 6 months after completion of chemotherapy, was similar between patients in the control and docetaxel groups of the safety population who had not already progressed; AE data collection stopped at disease progression. Quality of life is an important factor in treatment decisions; it is reassuring there was no evidence of persistent toxicity associated with worse quality of life. Our findings are consistent with studies that demonstrated quality of life can improve back to baseline after chemotherapy (22-24). The AEs associated with long-term hormone therapy in both groups remain considerable.

Our analysis confirms the FFS benefit associated with this approach, with clear evidence that patients treated with upfront docetaxel lived longer without their disease relapsing. This would also mean that men could continue their lives for a longer period without the need for additional therapeutic intervention. This in turn may augment any psychological benefit arising from living without signs of disease progression, which is considered important for many patients. Participant-reported quality-of-life measures are an important contributor to quantifying treatment impact, although these data are typically less well recorded in clinical trials after disease progression. We previously demonstrated, consistent with this, how upfront docetaxel increased quality-adjusted life-years in this group of nonmetastatic patients (25). Examining participant-reported quality-of-life measures may more accurately quantify treatment impact, although these data are less well recorded after disease progression.

Our findings align with results from other trials of docetaxel in nonmetastatic prostate cancer (2,26,27). These trials had subtle differences in inclusion criteria, treatment regimens, and outcome measures, yet most found good evidence that docetaxel increased FFS and PFS and insufficient evidence that it prolonged time to metastases or death. The analysis of pooled, aggregate data showed some evidence that docetaxel may improve overall survival in nonmetastatic prostate cancer. This may reflect a subset effect; reflect a small, broad effect; or be chance alone. Further exploration may be warranted through individual patient data meta-analysis. However, these findings on overall survival were not well powered with fewer than 500 deaths reported across the eligible trials. An individual randomized controlled trial planned to look for a hazard ratio of 0.85 with a 2-sided alpha of 0.05, 80% power and equal allocation ratio would have required sufficient recruitment and follow-up for the reporting of approximately 1000 deaths.

During recruitment to STAMPEDE’s docetaxel comparison, recommendations for treating nonmetastatic patients with local radiotherapy were adapted in response to then emerging results from other trials (14,15), which showed prostate radiotherapy improved overall and disease-specific survival when combined with ADT in high-risk, nonmetastatic patients without known nodal involvement. Thus in 2011, radical prostate radiotherapy became part of SOC for patients with N0 nonmetastatic disease. Radiotherapy for patients with N+ nonmetastatic disease remained at the treating clinician’s discretion. The planned use of radiotherapy was collected at baseline to balance across treatment groups, although radiotherapy was started later in the chemotherapy group, after docetaxel.

Our exploratory analysis of this SOC radiotherapy’s impact in nonmetastatic patients, regardless of allocation to the control or docetaxel group, found good evidence radiotherapy improved FFS and some evidence of improved PFS. This was consistent across N0 and N+ patients, albeit more prominently for N0 patients. We could also explore any interaction between radiotherapy and docetaxel. There was some evidence that SOC radiotherapy’s benefit was apparent in the control group but not the docetaxel group (ie, there was no evidence of additive benefit from using both radiotherapy and docetaxel). Clinicians should consider this information carefully when making treatment decisions with nonmetastatic patients.

Overall, these long-term analyses of nonmetastatic patients in STAMPEDE did not demonstrate a benefit to using docetaxel chemotherapy in terms of metastasis-free survival or overall survival. There was good evidence that upfront docetaxel resulted in men living longer before their disease relapsed, and good evidence that there was no excess of long-term AEs for these patients. The findings are consistent with trials addressing the same broad question and provide some evidence of modest benefit in favor of chemotherapy. These points will be worth considering altogether for selected men in this population and interpreted in the context of more recent data reporting a large statistically significant and clinically meaningful benefit for adding abiraterone acetate and prednisolone in this population in the same STAMPEDE protocol (28). The benefits of SOC radiotherapy in patients not having docetaxel were confirmed. The data suggest that, for patients planned for radical radiotherapy, upfront chemotherapy can be avoided.

## Funding

This work was supported by Cancer Research UK (grant number CRUK_A12459); Medical Research Council (grant number MRC_MC_UU_12023/25, grant number MC_UU_00004/01); Sanofi; Astellas; Clovis; Janssen; Novartis; Pfizer. NDJ, CCP, and DPD were supported by the National Institute for Health Research (NIHR) Biomedical Research Centre at The Royal Marsden NHS Foundation Trust and the Institute of Cancer Research, London.

## Notes

**Role of the funders:** The study funders had no role in the design of the study; the collection, analysis, and interpretation of the data; the writing of the manuscript; or the decision to submit the manuscript for publication.

**Disclosures:** GA: Honoraria (self), Advisory/Consultancy, Speaker Bureau/Expert testimony, Travel/Accommodation/Expenses, Non-remunerated activity/ies: Astellas; Advisory/Consultancy, Travel/Accommodation/Expenses, Non-remunerated activity/ies: Medivation; Advisory/Consultancy: Novartis; Advisory/Consultancy: Millennium Pharmaceuticals; Advisory/Consultancy, Travel/Accommodation/Expenses, Non-remunerated activity/ies: Abbott Laboratories; Advisory/Consultancy, Travel/Accommodation/Expenses, Non-remunerated activity/ies: Essa Pharmaceuticals; Advisory/Consultancy, Travel/Accommodation/Expenses, Nonremunerated activity/ies: Bayer Healthcare Pharmaceuticals; Speaker Bureau/Expert testimony: Takeda; Speaker Bureau/Expert testimony: Sanofi-Aventis; Research grant/Funding (self): AstraZeneca; Research grant/Funding (self): Arno Therapeutics; Research grant/Funding (self): Innocrin Pharma; Honoraria (self), Advisory/Consultancy, Speaker Bureau/Expert testimony, Research grant/Funding (self), Travel/Accommodation/Expenses, Non-remunerated activity/ies: Janssen; Advisory/Consultancy: Veridex; Advisory/Consultancy, Speaker Bureau/Expert testimony, Travel/Accommodation/Expenses, Non-remunerated activity/ies: Roche/Ventana; Advisory/Consultancy, Non-remunerated activity/ies: Pfizer; Research grant/Funding (self), employee of the Institute of Cancer Research (ICR), where abiraterone acetate was developed, up to January 8, 2018, ICR. AJB: Speaker fees: Janssen. Support for attending meetings and/or travel: Janssen; SC: Honoraria, Speakers fee, Travel Grant- Janssen Pharmaceutical. NWC: Advisory/Consultancy: Janssen. WC: Dr Cross reports personal fees from Janssen and other from Bayer outside the submitted work. DPD: Research grant/Funding (institution), Financial Support for Trial Recruitment: UK National Institute for Health Research Clinical Research Network (NIHR CRN); Research grant/Funding (institution): ICR; Research grant/Funding (self), C46/A3976, C46/A10588 and C33589/A19727: Cancer Research UK; Honoraria (self), Advisory/Consultancy, Speaker Bureau/Expert testimony: Takeda; Honoraria (self), Advisory/Consultancy, Speaker Bureau/Expert testimony: Amgen; Honoraria (self), Advisory/Consultancy, Speaker Bureau/Expert testimony: Astellas; Advisory/Consultancy, Travel/Accommodation/Expenses: Sandoz; Advisory/Consultancy, Speaker Bureau/Expert testimony, Travel/Accommodation/Expenses: Janssen. R. Jones: Honoraria (self), Advisory/Consultancy, Speaker Bureau/Expert testimony: Janssen; Honoraria (self), Advisory/Consultancy, Speaker Bureau/Expert testimony, Research grant/Funding (self): Astellas; Honoraria (self), Advisory/Consultancy, Speaker Bureau/Expert testimony, Research grant/Funding (self): Sanofi; Honoraria (self), Advisory/Consultancy, Speaker Bureau/Expert testimony: Novartis. SG: Honorarium payments to hospital- Roche, Innocrin Pharmaceuticals, Sanofi, Novartis, Cell Search, Clovis and Bristol-Myers Squibb. Uncompensated advisory role- Nectar Therapeutic and ProteoMedix. NDJ: Advisory/Consultancy: Sanofi; Advisory/Consultancy: Novartis; Advisory/Consultancy, Speaker Bureau/Expert testimony, Research grant/Funding (self), Travel/Accommodation/Expenses, Non-remunerated activity/ies: Janssen. RJJ: Grants or contracts paid to my institution from Bayer plc, Pfizer and Astellas. Consulting fees—Janssen Advisory board honoraria, Astellas Advisory board honoraria, consultancy, Bayer Advisory board honoraria, Pfizer Advisory board honoraria. Payment or honoraria for lectures, presentations, speakers bureaus, manuscript writing or educational events- speakers fees from Janssen, Astellas, Bayer, Pfizer. AM: Research grants from Cancer Research UK’s Clinical Research Committee, from Medical Research Council, from Novartis, from Sanofi-Aventis, from Pfizer, from Janssen Pharma NV, from Astellas, from Clovis Oncology, during the conduct of the study. MDM: Honoraria (self), Speaker Bureau/Expert testimony: Sanofi; Speaker Bureau/Expert testimony: Janssen; Speaker Bureau/Expert testimony: Bayer. ZIM: Advisory boards- Sanofi, Janssen and Pfizer. Assisted travel to Congress- Bayer. JOS: Payment or honoraria for lectures, presentations, speakers bureaus, manuscript writing or educational events; AAA, Astellas, Bayer, Janssen, Novartis, Sanofi. Participation on a Data Safety Monitoring Board or Advisory Board; AAA, Astellas, Bayer, Janssen, Novartis, Sanofi. CCP: Dr Parker reports grants, personal fees and other from Bayer, other from AAA and personal fees from Janssen, outside the submitted work. MKBP: Research grant/Funding (self), Unrestricted grant to contribute to STAMPEDE overall: Astellas; Research grant/Funding (self), Unrestricted grant to contribute to STAMPEDE overall: Clovis Oncology; Research grant/Funding (self), Unrestricted grant to contribute to STAMPEDE overall: Novartis; Research grant/Funding (self), Unrestricted grant to contribute to STAMPEDE overall: Pfizer; Research grant/Funding (self), Unrestricted grant to contribute to STAMPEDE overall: Sanofi. SS: Payment or honoraria for lectures, presentations, speaker’s bureaus, manuscript writing or educational events, from Bayer, Clovis Oncology, Pfizer. Participation on a Data Safety Monitoring Board or Advisory Board; Bayer. Support for attending meetings and/or travel: BMS, Roche. MRC: Research grant/Funding (self), Non-remunerated activity/ies, Unrestricted grant to contribute to STAMPEDE overall: Astellas; Research grant/Funding (self), Non-remunerated activity/ies, Unrestricted grant to contribute to STAMPEDE overall: Clovis Oncology; Research grant/Funding (self), Non-remunerated activity/ies, Unrestricted grant to contribute to STAMPEDE overall: Novartis; Research grant/Funding (self), Non-remunerated activity/ies, Unrestricted grant to contribute to STAMPEDE overall: Pfizer; Speaker Bureau/Expert testimony, Travel/Accommodation/Expenses: Eli Lilly; Speaker Bureau/Expert testimony, Research grant/Funding (self), Travel/Accommodation/Expenses, Non-remunerated activity/ies, Unrestricted grant to contribute to STAMPEDE overall: Janssen; Research grant/Funding (self), Non-remunerated activity/ies, Unrestricted grant to contribute to STAMPEDE overall: Sanofi. JT: Participation on a Data Safety Monitoring Board or Advisory Board: Astra Zeneca, Astellas, Bayer. Support for attending meetings and/or travel: Jansen, Roche, Bayer; JW: Payment or honoraria for lectures, presentations, speakers bureaus, manuscript writing or educational events: Bristol Myers Squibb, MSD, Eisai, Novartis. All other authors did not declare a conflict of interest.

**Author contributions:** Conceptualization: MKBP, NDJ, MRS, REL, NWC, MDM, DPD. Data curation: All. Formal Analysis: FI, AC, MRS. Funding acquisition: MKBP, NDJ, MRS, NWC, MDM, DPD, SG. Investigation: All. Methodology: MKBP, FI, AC, MRC. Project administration: MKBP, CA, REL, MRS, HR. Resources: All. Software: FI, AC, MRC. Supervision: NDJ, FI, NWC, CA, GA, CDB, SC, WC, DPD, DCG, SG, RJJ, REL, ZIM, MDM, DJM, RM, CCP, HLR, JMR, MKBP, MRS. Validation: FI, AC, MRS. Visualization: FI, AC, MRS. Writing - original draft: NDJ, FI, HR, MRS. Writing - review & editing: All.

**Acknowledgements:** We recognize the efforts of all trial team members at the trials units and hospitals who have supported and engaged with STAMPEDE. Investigators and oversight committee members are listed in the Supplementary Materials (available online). Mahesh Parmar originated the MAMS concept. Nicholas James was the chief investigator. We thank Laura Murphy and Tim Morris for putting the time-to-event graphs into KMunicate format (20). We thank Jayne Tierney, David Fisher, Sarah Burdett, and the STOPCAP team for comments on the combined analysis. Finally, and most importantly, we recognize and thank all of the participants of the trial and the families and friends who supported them. Clinical trials happen only because people choose to join them.

## Supplementary Material

pkac043_Supplementary_Data

## Data Availability

The data underpinning these analyses are available upon request for an appropriate data reused project as per the moderated access approach of MRC CTU at UCL: https://www.ctu.mrc.ac.uk/our-research/other-research-policy/data-sharing/. Please contact the corresponding author for more information via mrcctu.datareleaserequest@ucl.ac.uk.
